# Primary tumour response on breast MRI as a predictor of axillary pathologic response in breast cancer patients treated with neoadjuvant chemotherapy

**DOI:** 10.1007/s00330-025-12249-x

**Published:** 2025-12-23

**Authors:** Florien J. G. van Amstel, Rik G. M. van Mierlo, Patty J. Nelemans, Sanne M. E. Engelen, Janneke Houwers, Loes F. S. Kooreman, Vivianne C. G. Tjan-Heijnen, Sabine Siesling, Marjolein L. Smidt, Thiemo J. A. van Nijnatten

**Affiliations:** 1 Department of Radiology and Nuclear Medicine, Maastricht University Medical Centre+, Maastricht, The Netherlands; 2 GROW–Research Institute for Oncology and Reproduction, Maastricht University, Maastricht, The Netherlands; 3 Department of Surgery, Maastricht University Medical Centre+, Maastricht, The Netherlands; 4 Department of Epidemiology, Maastricht University, Maastricht, The Netherlands; 5 Department of Pathology, Maastricht University Medical Centre+, Maastricht, The Netherlands; 6 Department of Medical Oncology, Maastricht University Medical Centre+, Maastricht, The Netherlands; 7 Department of Research and Development, Netherlands Comprehensive Cancer Centre, (IKNL), Utrecht, The Netherlands; 8 Department of Health Technology and Services Research, Technical Medical Centre, University of Twente, Enschede, The Netherlands

**Keywords:** Breast neoplasms, Lymph nodes, Neoadjuvant therapy, Magnetic resonance imaging

## Abstract

**Objectives:**

To investigate whether breast radiologic response on breast MRI can predict axillary pathologic response (ypN0 vs. ypN+) in breast cancer patients treated with neoadjuvant chemotherapy (NACT) in the total study population and in subgroups according to clinical nodal (cN)-status and oestrogen receptor (ER)-expression.

**Materials and methods:**

This retrospective single-centre study consecutively included breast cancer patients who, between 2012 and 2022, had undergone baseline and post-NACT breast MRI. Measures of predictive ability, including sensitivity, specificity, positive predictive value (PPV), negative predictive value (NPV), and diagnostic odds ratio (DOR) with 95% confidence intervals (CI), were calculated. Subgroup analyses were performed for cN-status and ER expression.

**Results:**

Of 251 included patients, 48.6% were cN0 and 34.7% had ER-negative tumours. For axillary pathologic response prediction by breast MRI, the sensitivity was 84%, specificity 40.4%, PPV 48.3%, NPV 79.2%, and DOR 3.6 (95% CI: 1.83–7.00). The probability of achieving ypN0 was 60.2% in all patients, 82% in cN0 patients versus 39.5% in cN+ patients, and 78.2% in ER-negative tumours versus 50.6% in ER-positive tumours. High NPVs were observed in cN0 patients (92%) and ER-negative tumours (90.1%) and were lower in cN+ patients (62.2%) and ER-positive tumours (72.2%).

**Conclusions:**

The probability of ypN+ given breast complete response on breast MRI was below 10% in cN0 patients and ER-negative tumours. Therefore, in these subgroups, breast complete response on breast MRI may be clinically useful to exclude ypN+. In cN+ patients and ER-positive tumours, due to lower NPVs, breast complete response on breast MRI may not be clinically useful to exclude ypN+.

**Key Points:**

***Question**** Accurate non-invasive methods to differentiate between patients with and without axillary pathologic complete response after neoadjuvant chemotherapy prior to axillary surgery are currently lacking*.

***Findings**** Breast complete response on MRI after neoadjuvant chemotherapy indicates a low probability (< 10%) of axillary residual disease in clinically node-negative patients and oestrogen receptor-negative tumours*.

***Clinical relevance**** Breast complete response on MRI may be a non-invasive, clinically useful tool to exclude patients with axillary residual disease after neoadjuvant chemotherapy prior to axillary surgery and guide patient selection for potential de-escalation of axillary treatment*.

**Graphical Abstract:**

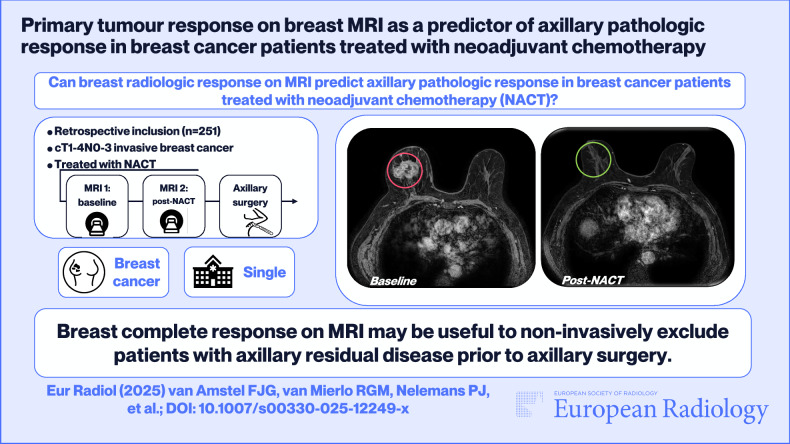

## Introduction

In recent years, the use of systemic therapy in breast cancer treatment has shifted from adjuvant to neoadjuvant chemotherapy (NACT) [1, 2]. During NACT, treatment response is often monitored with breast MRI, which provides the most accurate information regarding the response of the primary tumour [3, 4]. Complete disappearance of the primary tumour with no pathological (non-mass) parenchymal enhancement on breast MRI is referred to as breast radiologic complete response (rCR). Previous studies have demonstrated both surgical and prognostic benefits of breast rCR on breast MRI after NACT [5–7].

In addition, accurate monitoring of axillary lymph node response to NACT prior to axillary surgery is crucial to guide patient selection for potential de-escalation of axillary treatment. However, the response evaluation of axillary lymph node metastases with breast MRI remains challenging. While the axillary region is generally included in the breast MRI, it is not consistently fully visualised in all breast MRI exams due to limitations in patient positioning, coverage area, and protocol differences, which limit the diagnostic performance of breast MRI in assessing axillary lymph node response [8–10].

Following NACT, tumour cells can be completely eradicated at the time of surgery, which is called pathologic complete response of the breast (ypT0) and/or axillary lymph nodes (ypN0) [11]. Pathologic complete response after NACT is related to improved overall long-term disease-free and overall survival [12]. Various studies observed that ypT0 is associated with ypN0 after NACT [12–15]. A key limitation is that pathology results can only be evaluated after breast and axillary surgery following NACT. Accordingly, one might wonder whether breast radiologic response on breast MRI can provide valuable information to predict axillary pathologic response preoperatively.

Importantly, the probability of ypN0 after NACT varies among patient subgroups, depending on clinical nodal (cN) status and breast cancer subtypes. A study by de Wild et al showed that 81.1% of clinically node-negative (cN0) patients versus 36.6% of clinically node-positive (cN+) patients achieved ypN0 after NACT [16]. In addition, ypN0 rates of 18% for hormone receptor (HR)-positive/human epidermal growth factor receptor 2 (HER2)-negative, 45% for HR-positive/HER2-positive, 60% for HR-negative/HER2-positive, and 48% for triple-negative patients were found in cN+ patients after NACT [11]. In patient subgroups with a high probability of ypN0 after NACT, breast radiologic response on breast MRI may be clinically useful for the prediction of ypN0, which could potentially guide de-escalation strategies in axillary treatment.

First, this study investigated whether breast radiologic response on breast MRI can predict axillary pathologic response in breast cancer patients treated with NACT. Second, the ability of breast radiologic response to predict axillary pathologic response in subgroups of patients according to cN-status and oestrogen receptor (ER) expression was assessed.

## Materials and methods

### Study design and study population

This retrospective single-centre study was approved by the local medical ethics committee, which waived the necessity to obtain written informed consent (reference number: 2022-3481). Data were collected from medical records of female patients diagnosed with invasive breast cancer (cT1-4N0-3) and treated with NACT between January 1, 2012, and December 31, 2022, at Maastricht University Medical Centre+ (Maastricht UMC+, The Netherlands).

**Fig. 1:**
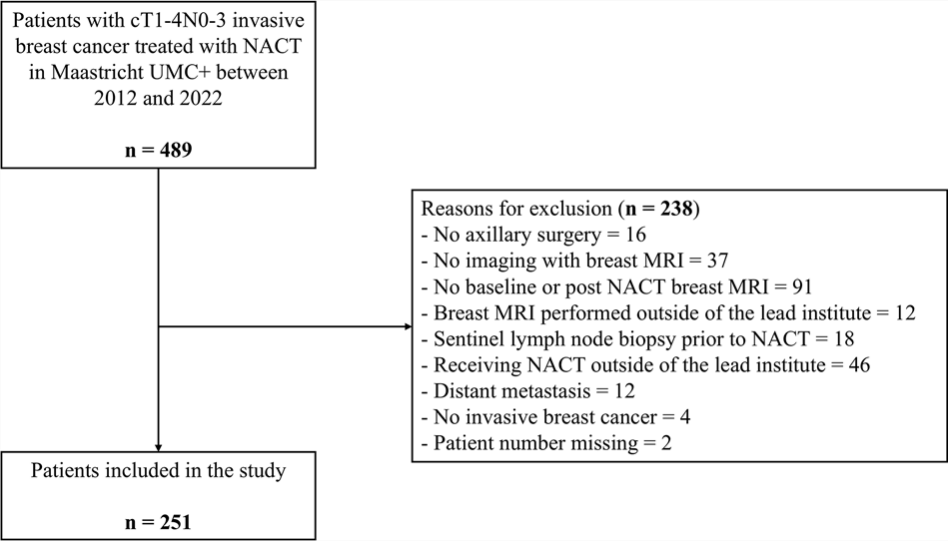
Flowchart of patient inclusion and exclusion. NACT, neoadjuvant chemotherapy

Consecutively included were all patients who underwent baseline and post-NACT imaging with breast MRI followed by axillary surgery. Patients were excluded in case of (oligo) distant metastases, endocrine therapy or radiotherapy before NACT, and NACT or imaging performed outside of the lead institute. Figure 1 illustrates the derivation of the final study population.

### Breast radiologic response

Breast radiologic response to NACT was derived from reports on the assessment of breast MRI exams that had been performed at baseline and post-NACT. The breast MRI exams had been conducted in prone position on two different 1.5-Tesla MRI scanners (Ingenia and Intera, Philips Healthcare), using a dedicated 16-channel breast coil. During the study period, a standard pre- and post-gadolinium breast MRI protocol was used within Maastricht UMC+. The standard breast MRI protocol consisted of nonenhanced 3D T2W Turbo Spin Echo sequence and dynamic, contrast-enhanced T1W sequence protocols using fat saturation and diffusion weighted imaging. Over the years, only slight adjustments were made to the breast MRI protocol (Table S1).

**Fig. 2:**
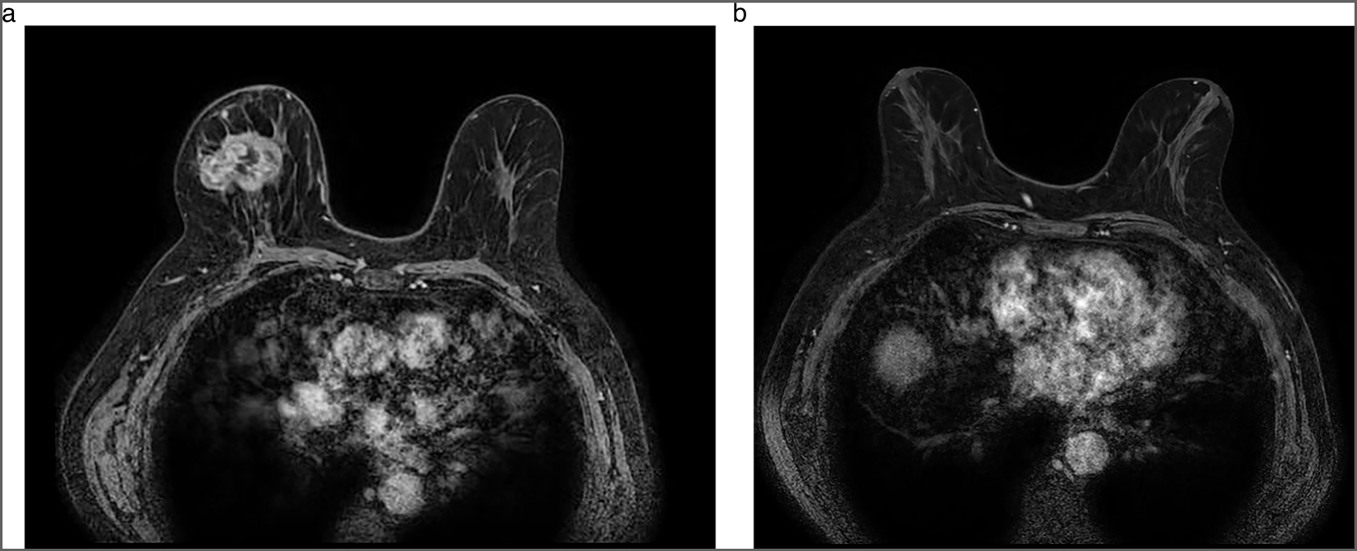
Example of a 77-year-old woman diagnosed with a right unifocal invasive breast carcinoma grade 3 with a triple-negative subtype and biopsy-proven axillary lymph node metastases (cN1). Baseline breast MRI prior to NACT demonstrated a 47 mm large mass (**a**). Post-NACT breast MRI demonstrated the achievement of a breast radiologic complete response (**b**). Following NACT and surgery, the patient achieved ypT0N0. NACT, neoadjuvant chemotherapy

A negative result was defined as breast rCR, characterised by the absence of any residual signs of malignancy within the breast on breast MRI, according to Breast Imaging-Reporting and Data System of the American College of Radiology (BI-RADS) lexicon [17] (Fig. 2). Cases in which the breast radiologist reported that breast rCR was possible (i.e., either rCR or near rCR within the breast) were also classified as breast rCR. A positive test was defined as breast radiologic residual disease (rRD), indicating the presence of any residual enhancement on breast MRI.

### Evaluation of axillary pathologic response

Information about ypN-status was derived from pathological reports on findings during axillary surgery and served as the reference standard. ypN0 was defined as the absence of any axillary residual disease, including isolated tumour cells, and ypN+ was defined as the presence of residual disease.

### Classification into subgroups

Information on the presence of axillary lymph node metastases prior to NACT (cN) was retrieved from patient medical records. In all cases, cN-status had been determined by axillary ultrasound and fine needle aspiration cytology or core needle biopsy in case axillary ultrasound showed suspicious axillary lymph nodes. The expression of ER and HER2 was noted from histopathologic reports of core biopsies, which had been performed at baseline. A percentage of ≥ 10% tumour cells with core-staining was defined as positive ER expression; other results were classified as negative in concordance with Dutch national guidelines. The HER2 status had been evaluated with fluorescence in situ hybridisation and immunohistochemistry if necessary [18–20]. In case of multifocal tumours, the tumour with the highest receptor expression was reported.

### Neoadjuvant chemotherapy

During the study period, Dutch national breast cancer guidelines of 2012 and 2020 were applied [21]. Neoadjuvant chemotherapy consisted of anthracycline- and/or taxane-based chemotherapy, with or without carboplatin. Patients with HER2-positive breast cancer received additional targeted therapy (trastuzumab alone or trastuzumab and pertuzumab).

### Statistical analysis

Positive predictive value (PPV) and negative predictive value (NPV) were used to evaluate the predictive value of breast radiologic response on breast MRI for axillary pathologic response. Discriminative ability was expressed as sensitivity, specificity, and diagnostic odds ratios (DOR) with 95% confidence intervals (CI). For subgroup analyses, patients were categorised into four subgroups: cN0 and cN+ patients and ER-negative and ER-positive tumours. For each subgroup, calculation of predictive values (PPV and NPV) was based on sensitivity, specificity, and probability of ypN0 in the subgroup. In addition, a sensitivity analysis was performed using the sensitivity and specificity of the total population. This analysis leads to results that are less subject to sampling variation, but it assumes that sensitivity and specificity do not differ between subgroups. Statistical analyses were performed using IBM SPSS Statistics (version 26.0, IBM Corp.) and StatPages.net (Bernard Rosner, Fundamentals of Biostatistics, 6th edn, 2006).

## Results

### Baseline and treatment characteristics

Of the 251 included patients, the median age was 53 years. A total of 48.6% (122/251) patients were classified as cN0, and primary tumours were ER-negative in 34.7% (87/251) of the patients. Breast MRI showed 30.7% (77/251) patients with breast rCR. After axillary surgery, 60.2% (151/251) of the patients had ypN0, 82% (100/122) in cN0 patients versus 39.5% (51/129) in cN+ patients, and 78.2% (68/87) in ER-negative tumours versus 50.6% (83/164) in ER-positive tumours. Baseline and treatment characteristics of included patients are listed in Table 1.

**Table 1:** Baseline and treatment characteristics

Variables	Overall (*n* = 251)		cN0 (*n* = 122)		cN + (*n* = 129)		ER-positive (*n* = 164)		ER-negative (*n* = 87)	
Age (years)										
Median [IQR]	53	[46–60]	52.5	[47–59]	53	[46–61]	52.5	[46–60]	53	[43–60]
Clinical T-status										
cT1	42	(16.7%)	25	(20.5%)	17	(13.2%)	29	(17.7%)	13	(14.9%)
cT2	149	(59.4%)	80	(65.6%)	69	(53.5%)	102	(62.2%)	47	(54%)
cT3	42	(16.7%)	13	(10.7%)	29	(22.5%)	25	(15.2%)	17	(19.6%)
cT4	18	(7.2%)	4	(3.3%)	14	(10.8%)	8	(4.9%)	10	(11.5%)
Clinical N-status										
cN0	122	(48.6%)	—	—	—	—	78	(47.6%)	44	(50.6%)
cN1	99	(39.4%)	—	—	—	—	75	(45.7%)	24	(27.6%)
cN2	7	(2.8%)	—	—	—	—	3	(1.8%)	4	(4.6%)
cN3	23	(9.2%)	—	—	—	—	8	(4.9%)	15	(17.2%)
Tumour histology										
No special type	223	(88.8%)	109	(89.3%)	114	(88.4)	143	(87.2)	80	(92%)
Lobular	17	(6.8%)	9	(7.4%)	8	(6.2)	17	(10.4)	0	(0%)
Other	11	(4.4%)	4	(3.3%)	7	(5.4)	4	(2.4)	7	(8%)
Tumour grade										
1	12	(4.8%)	5	(4.1%)	7	(5.4%)	12	(7.3%)	0	(0%)
2	115	(45.8%)	53	(43.4%)	62	(48.1%)	96	(58.5%)	19	(21.9%)
3	110	(43.8%)	59	(48.4%)	51	(39.5%)	49	(29.9%)	61	(70.1%)
Unknown	14	(5.6%)	5	(4.1%)	9	(7%)	7	(4.3%)	7	(8%)
ER										
Negative	87	(34.7%)	44	(36.1%)	43	(33.3%)	—	—	—	—
Positive	164	(65.3%)	78	(63.9%)	86	(66.7%)	—	—	—	—
HER2										
Negative	178	(70.9%)	86	(70.5%)	92	(71.3%)	119	(72.6%)	59	(67.8%)
Positive	73	(29.1%)	36	(29.5%)	37	(28.7%)	45	(27.4%)	28	(32.2%)
Clinical subtype										
ER+/HER2-	120	(47.8%)	55	(45.1%)	65	(50.4%)	—	—	—	—
ER+/HER2+	44	(17.5%)	23	(18.9%)	21	(16.3%)	—	—	—	—
ER-/HER2+	28	(11.2%)	12	(9.8%)	16	(12.4%)	—	—	—	—
Triple negative	59	(23.5%)	32	(26.2%)	27	(20.9%)	—	—	—	—
Axillary surgery										
SLNB	122	(48.6%)	115	(94.3%)	7	(5.4%)	77	(47%)	45	(51.7%)
MARI-procedure	2	(34.3%)	0	(0%)	2	(1.6%)	2	(1.2%)	0	(0%)
RISAS-procedure	41	(0.8%)	3	(2.4%)	38	(29.4%)	26	(15.8%)	15	(17.3%)
ALND	86	(16.3%)	4	(3.3%)	82	(63.6%)	59	(36%)	27	(31%)

“—” means “not applicable”

*cN0* clinically node-negative, *cN+* clinically node-positive, *ER* oestrogen receptor, *IQR* interquartile range, *HER2* human epidermal growth factor receptor 2, *SLNB* sentinel lymph node biopsy, *MARI-procedure* marking axillary lymph node with radioactive iodine seed, *RISAS-procedure* radioactive iodine seed placement in the axilla with sentinel lymph node biopsy, *ALND* axillary lymph node dissection

### Breast rCR on breast MRI as a predictor of ypN0

The probability of ypN0 as well as the sensitivity, specificity, PPV, and NPV in the total population and stratified by cN-status and ER expression are presented in Tables 2 and 3. In the total population, the probability of ypN0 in case of breast rCR (NPV) was 79.2% (61/77). Using the subgroup-specific sensitivity and specificity values, the NPV was 85.4% (35/41) in cN0 patients, 72.2% (26/36) in cN+ patients, 96.8% (30/31) in ER-negative tumours, and 67.4% (31/46) in ER-positive tumours. If the sensitivity and specificity in the total population were used for the calculation of predictive values, the NPV remained high in both cN0 patients and ER-negative tumours (92% and 90.1%, respectively) and remained moderate in both cN+ patients and ER-positive tumours (62.2% and 72.2%, respectively).

**Table 2:** Diagnostic performance of breast MRI in the total study population

Overall						
		ypN+	ypN0	Total		
	Breast rRD	84	90	174	ypN0	60.2% (151/251)
	Breast rCR	16	61	77	Sensitivity	84% (84/100)
	Total	100	151	251	Specificity	40.4% (61/151)
					DOR	3.6 (95% CI: 1.83–7.00)
					PPV	48.3% (84/174)
					NPV	79.2% (61/77)

*ypN+* pathologic residual disease, *ypN0* absence of axillary lymph node metastases, *rRD* radiologic residual disease, *rCR* radiologic complete response, *DOR* diagnostic odds ratio, *CI* confidence interval, *PPV* positive predictive value, *NPV* negative predictive value

**Table 3:** Diagnostic performance of breast MRI stratified by cN-status and ER expression

cN0						
		ypN+	ypN0	Total		
	Breast rRD	16	65	81	ypN0	82% (100/122)
	Breast rCR	6	35	41	Sensitivity	72.7% (16/22)
	Total	22	100	122	Specificity	35% (35/100)
					DOR	1.4 (95% CI: 0.47–4.55)
					PPV	19.8% (16/81)
					NPV	85.4% (35/41)
					Corrected PPV*	23.6%
					Corrected NPV*	92%

cN+						
		ypN+	ypN0	Total		
	Breast rRD	68	25	93	ypN0	39.5% (51/129)
	Breast rCR	10	26	36	Sensitivity	87.2% (68/78)
	Total	78	51	129	Specificity	51% (25/51)
					DOR	7.1 (95% CI: 2.77–18.44)
					PPV	73.1% (68/93)
					NPV	72.2% (26/36)
					Corrected PPV*	68.3%
					Corrected NPV*	62.2%

ER-negative						
		ypN+	ypN0	Total		
	Breast rRD	18	38	56	ypN0	78.2% (68/87)
	Breast rCR	1	30	31	Sensitivity	94.7% (18/19)
	Total	19	68	87	Specificity	44.1% (30/68)
					DOR	14.2 (95% CI: 1.81–301.73)
					PPV	32.1% (18/56)
					NPV	96.8% (30/31)
					Corrected PPV*	28.2%
					Corrected NPV*	90.1%

ER-positive						
		ypN+	ypN0	Total		
	Breast rRD	66	52	118	ypN0	50.6% (83/164)
	Breast rCR	15	31	46	Sensitivity	81.5% (66/81)
	Total	81	83	164	Specificity	37.3% (31/83)
					DOR	2.6 (95% CI: 1.21–5.72)
					PPV	55.9% (66/118)
					NPV	67.4% (31/46)
					Corrected PPV*	57.9%
					Corrected NPV*	72.2%

*cN0* clinically node-negative, *cN+* clinically node-positive, *ER* oestrogen receptor, *ypN+* pathologic residual disease, *ypN0* absence of axillary lymph node metastases, *rRD* radiologic residual disease, *rCR* radiologic complete response, *DOR* diagnostic odds ratio, *CI* confidence interval, *PPV* positive predictive value, *NPV* negative predictive value

* Corrected PPV and NPV were calculated using the sensitivity and specificity values of the total population, combined with the subgroup-specific prevalence of ypN0

### Breast rRD on breast MRI as a predictor of ypN+

In the total population, the probability of ypN+ in case of breast rRD (PPV) was 48.3% (84/174) (Table 2). PPV was higher in cN+ patients (73.1%, 68/93) and ER-positive tumours (55.9%, 66/118), with the highest probability of ypN+ after NACT. When using the sensitivity and specificity estimates in the total population, the PPV was 68.3% in cN+ patients and 57.9% in ER-positive tumours (Table 3).

### Diagnostic odds ratio

The DOR combines sensitivity and specificity and was used as a single indicator for the ability of breast radiologic response assessment on breast MRI to predict axillary pathologic response. The DOR in the total population was 3.6 (95% CI: 1.83–7.00). In the four subgroups, the DOR fluctuated between 1.4 (95% CI: 0.47–4.55) in cN0 patients to 14.2 (95% CI: 1.81–301.73) in ER-negative tumours (Tables 2 and 3).

## Discussion

This retrospective single-centre study investigated whether breast radiologic response on breast MRI can predict axillary pathologic response after NACT. In this study, breast radiologic response on breast MRI had a sensitivity of 84% and a specificity of 40.4% for the prediction of axillary pathologic response. In subgroups of cN0 patients and patients with ER-negative tumours, with a high probability of ypN0 after NACT, the NPV was 92% and 90.1%, respectively. The probability that ypN+ was present in the case of breast rCR on breast MRI was lower than 10% in these subgroups. In contrast, subgroups of cN+ patients and patients with ER-positive tumours demonstrated lower NPVs of 62.2% and 72.2%, reflecting their lower probability of ypN0 after NACT. The probability that ypN+ was present in the case of breast rCR on breast MRI was higher than 25% in these subgroups. The PPV in the total population was 48.3% and slightly higher in subgroups of cN+ patients (68.3%) and patients with ER-positive tumours (57.9%).

Due to the frequent use and advancements in NACT regimens, ypN0 rates have significantly increased over the past decade [22, 23]. To guide patient selection for potential de-escalation of axillary treatment, accurate response monitoring to NACT is crucial to differentiate between patients with ypN0 and ypN+ prior to axillary surgery. For breast MRI to have an added value in clinical practice for guiding de-escalation of axillary treatment, it is important that the risk of ypN+ in case of breast rCR on breast MRI remains as low as possible. Therefore, a high sensitivity and high NPV are needed.

The findings that in cN0 patients and patients with ER-negative tumours, the NPV of breast rCR is higher than 90% support the potential role of breast rCR diagnosis in guiding de-escalation of axillary treatment in these subgroups. Although axillary management in cN0 patients is already conservative, ongoing trials, such as the ASICS (NCT04225858) and NeoNAUTILUS (NCT06704945), are exploring whether sentinel lymph node biopsy can be safely omitted in selected cN0 patients with excellent response to NACT on imaging [24, 25]. In this context, this study provides retrospective evidence on the diagnostic performance of breast rCR assessment on breast MRI in cN0 patients, offering supportive data for these ongoing de-escalation efforts.

In cN+ patients, axillary management after NACT remains more complex. Less invasive surgical restaging procedures, such as targeted axillary dissection, have been implemented following NACT to reduce surgical morbidity related to axillary lymph node dissection [23, 26]. The pathology outcomes of these less invasive surgical restaging procedures remain essential for determining adjuvant axillary treatment [8]. However, despite the shift toward less invasive surgical restaging, the optimal axillary treatment strategy after NACT is still a topic of debate, particularly regarding whether axillary surgery can be safely omitted in selected cN+ patients [27]. Further de-escalation based on imaging modalities alone would require accurate preoperative prediction of response to NACT, as undertreatment in this subgroup (i.e., leaving behind therapy-resistant residual disease in unresected axillary lymph nodes) may give rise to later recurrence [28]. In this study, an NPV of only 62.2% was found, highlighting that breast rCR assessment on breast MRI alone seems insufficient to guide clinical decision making on axillary treatment de-escalation in this subgroup.

Nevertheless, accurate assessment of breast rCR on breast MRI remains essential, particularly since the diagnostic performance of breast MRI is not consistent across all patient subgroups. This study shows that the sensitivity and specificity of breast radiologic response differed between subgroups of cN0 and cN+ patients and patients with ER-negative and ER-positive tumours. These findings may be due to random fluctuation because of the relatively small sample size of the subgroups.

This study observed a higher sensitivity in cN+ (87.2%) compared to cN0 patients (72.7%), although there is no reason to assume that the assessment of breast radiologic response on breast MRI varies between cN0 and cN+ patients, as nodal status pertains to the axillary region and not to the breast. Similarly, Browne et al concluded that cN-status was not significantly associated with the accuracy of breast MRI in predicting post-NACT breast tumour size [29].

However, previous studies have reported that the diagnostic performance of breast MRI varies across tumours with different ER expression due to differences in imaging characteristics [30–34]. For example, ER-positive tumours often present as non-mass enhancement, are less vascular, and tend to shrink into small foci after NACT, which can lower the sensitivity of breast MRI. In contrast, ER-negative tumours more often appear on breast MRI as well-defined masses with a clear oval or round tumour shape, smooth mass margin, and rim enhancement, improving the visibility of residual enhancement and typically resulting in higher sensitivity [33–35]. Similarly, this study observed differences in sensitivity on breast MRI, with a higher sensitivity of 94.7% in patients with ER-negative tumours compared to 81.5% in patients with ER-positive tumours.

This study has some limitations. First, the data was collected from a single centre. The findings strongly depend on the diagnostic performance of the dedicated breast radiologists who assessed breast radiologic response and the probability of ypN0 that was achieved in patients who were treated in Maastricht UMC+ and may not be generalisable to other hospitals. Importantly, the fact that breast MRI exams were not centrally reviewed may have led to subjectivity and inter-rater variability in the assessment of the breast radiologic response. Second, patients were classified into cN0 and cN+ subgroups because further stratification by cN-status was not feasible due to the limited number of cN2-3 cases. Third, patients were classified into ER-negative and ER-positive tumour subgroups instead of subgroups with different breast cancer subtypes. The reason was that the number of ER-negative/HER2-positive and triple-negative patients was relatively small. Fourth, axillary radiologic response on post-NACT breast MRI was not incorporated in the analysis. Consequently, complementary information on axillary pathologic response from axillary radiologic response on post-NACT breast MRI could not be evaluated.

In conclusion, this study demonstrated that the probability that ypN+ was present in the case of breast rCR on breast MRI was lower than 10% in cN0 patients and patients with ER-negative tumours. Therefore, in these subgroups, breast rCR on breast MRI may be clinically useful to exclude ypN+ and guide de-escalation of axillary treatment. In contrast, in cN+ patients and patients with ER-positive tumours, the probability that ypN+ was present in the case of breast rCR on breast MRI was higher than 25%, indicating that breast rCR on breast MRI may not be clinically useful to exclude ypN+ in these subgroups.

## Supplementary information

ELECTRONIC SUPPLEMENTARY MATERIAL

## Abbreviations

BI-RADS: Breast Imaging-Reporting and Data System of the American College of Radiology
CI: Confidence interval
cN+: Clinically node-positive
cN0: Clinically node-negative
DOR: Diagnostic odds ratio
ER: Oestrogen receptor
HER2: Human epidermal growth factor receptor 2
HR: Hormone receptor
MRI: Magnetic resonance imaging
NACT: Neoadjuvant chemotherapy
NPV: Negative predictive value
PPV: Positive predictive value
rCR: Radiologic complete response
rRD: Radiologic residual disease
ypN+: Axillary pathologic residual disease
ypN0: Axillary pathologic complete response
ypT0: Breast pathologic complete response
