# Publisher Correction: Primary tumour response on breast MRI as a predictor of axillary pathologic response in breast cancer patients treated with neoadjuvant chemotherapy

**DOI:** 10.1007/s00330-026-12508-5

**Published:** 2026-04-02

**Authors:** Florien J. G. van Amstel, Rik G. M. van Mierlo, Patty J. Nelemans, Sanne M. E. Engelen, Janneke Houwers, Loes F. S. Kooreman, Vivianne C. G. Tjan-Heijnen, Sabine Siesling, Marjolein L. Smidt, Thiemo J. A. van Nijnatten

**Affiliations:** 1 Department of Radiology and Nuclear Medicine, Maastricht University Medical Centre+, Maastricht, The Netherlands; 2 GROW–Research Institute for Oncology and Reproduction, Maastricht University, Maastricht, The Netherlands; 3 Department of Surgery, Maastricht University Medical Centre+, Maastricht, The Netherlands; 4 Department of Epidemiology, Maastricht University, Maastricht, The Netherlands; 5 Department of Pathology, Maastricht University Medical Centre+, Maastricht, The Netherlands; 6 Department of Medical Oncology, Maastricht University Medical Centre+, Maastricht, The Netherlands; 7 Department of Research and Development, Netherlands Comprehensive Cancer Centre, (IKNL), Utrecht, The Netherlands; 8 Department of Health Technology and Services Research, Technical Medical Centre, University of Twente, Enschede, The Netherlands

**Correction to: European Radiology**

10.1007/s00330-025-12249-x published online 23 December 2025

In the PDF of the original article, due to a typesetting error, the continuation of Table 3 starts with the incorrect sub-heading “**cN0**”. The correct sub-heading is “**ER-positive**”.

The original article has been corrected.

