# Supplementary material for: Primary tumour response on breast MRI as a predictor of axillary pathologic response in breast cancer patients treated with neoadjuvant chemotherapy
Source: Eur Radiol. 2025 Dec 23;36(5):3358–66. doi: 10.1007/s00330-025-12249-x (PMC13086894; doi:10.1007/s00330-025-12249-x)
Supplement: Supplementary file 1 — ELECTRONIC SUPPLEMENTARY MATERIAL [file 330_2025_12249_MOESM1_ESM.pdf]

Primary tumour response on breast MRI as a predictor of axillary pathologic response in breast cancer patients treated with neoadjuvant chemotherapy

ELECTRONIC SUPPLEMENTARY MATERIAL

Table S1. Overview of breast MRI protocols in Maastricht UMC+ between 2012 – 2022

MRI 1: Philips Intera 1.5T

	2012	2013	2014
Coil	Sense breast 16-channel	Sense breast 16-channel	Sense breast 16-channel
Matrix	384 x 384	384 x 384	384 x 384
Pixel spacing (mm)	0.959^2	0.959^2	0.959^2
Field of View (mm)	368^2	368^2	368^2
In-plane resolution (mm)	1.0^2	1.0^2	1.0^2
Repetition time (msec)	2000	2000	2000
Echo time (msec)	205	205	258
Flip angle (degrees)	90	90	90
Echo train length	80	80	96
Slice thickness (mm)	2.0	2.0	2.0

MRI 2: Philips Ingenia 1.5T

	2012	2013	2014	2015	2016	2017	2018	2019	2020	2021	2022
Coil	Sense breast 16-channel	Sense breast 16-channel	Sense breast 16-channel	Sense breast 16-channel	Sense breast 16-channel	Sense breast 16-channel	Sense breast 16-channel	Sense breast 16-channel	Sense breast 16-channel	Sense breast 16-channel	Sense breast 16-channel
Matrix	400 x 400	400 x 400	400 x 400	400 x 400	400 x 400	432 x 432	432 x 432	432 x 432	432 x 432	432 x 432	432 x 432
Pixel spacing (mm)	0.854^2	0.854^2	0.854^2	0.854^2	0.854^2	0.787^2	0.787^2	0.787^2	0.787^2	0.787^2	0.787^2
Field of View (mm)	342^2	342^2	342^2	341^2	341^2	340^2	340^2	340^2	340^2	340^2	340^2
In-plane resolution (mm)	0.9^2	0.9^2	0.9^2	1.0^2	1.0^2	1.0^2	1.0^2	1.0^2	1.0^2	1.0^2	1.0^2
Repetition time (msec)	2000	2000	2000	2000	2000	2000	2000	2000	2000	2000	2000
Echo time (msec)	218	218	218	215	222	222	222	221	221	222	222
Flip angle (°)	90	90	90	90	90	90	90	90	90	90	90
Echo train length	95	95	95	95	95	95	95	95	95	95	95
Slice thickness (mm)	2.0	2.0	2.0	2.0	2.0	2.0	2.0	2.0	2.0	2.0	2.0
